# Low frequency of *POLD1* and *POLE* exonuclease domain variants in patients with multiple colorectal polyps

**DOI:** 10.1002/mgg3.603

**Published:** 2019-03-02

**Authors:** Fadwa A. Elsayed, Carli M. J. Tops, Maartje Nielsen, Dina Ruano, Hans F. A. Vasen, Hans Morreau, Frederik J. Hes, Tom van Wezel

**Affiliations:** ^1^ Department of Pathology LUMC Leiden The Netherlands; ^2^ Department of Clinical Genetics LUMC Leiden The Netherlands; ^3^ Department of Gastroenterology LUMC Leiden The Netherlands

**Keywords:** colorectal cancer, colorectal polyposis, DNA polymerase, exonuclease domain, germline mutations, POLD1

## Abstract

**Background:**

Germline mutations affecting the exonuclease domains of *POLE* and *POLD1* predispose to colorectal adenomas and carcinoma. Here, we aimed to screen the exonuclease domains to find the genetic causes of multiple colorectal polyps in unexplained cases.

**Methods:**

Using a custom next‐generation sequencing panel, we sequenced the exonuclease domains of *POLE* and *POLD1* in 332 index patients diagnosed with multiple colorectal polyps without germline alteration in colorectal polyposis predisposing genes.

**Results:**

We identified two variants of unknown significance. One germline *POLD1* c.961G>A, p.(Gly321Ser) variant was found in two cases. The first patient was diagnosed with multiple polyps at age 35 and colorectal cancer (CRC) at age 37, with no known family history of CRC. The second patient was diagnosed with CRC at age 44 and cumulatively developed multiple polyps; this patient had two sisters with endometrial cancer who did not carry the variant. Furthermore, we identified a novel *POLD1* c.955 T>G, p.(Cys319Gly) variant in a patient diagnosed with multiple colorectal adenomas at age 40. Co‐segregation analysis showed that one sister who cumulatively developed multiple adenomas from age 34, and another sister who developed CRC at age 38 did not carry the variant. We did not identify pathogenic variants in *POLE* and *POLD1*.

**Conclusion:**

This study confirms the low frequency of causal variants in these genes in the predisposition for multiple colorectal polyps, and also establishes that these genes are a rare cause of the disease.

## 1 INTRODUCTION

The heritable component of colorectal cancer (CRC) is approximately 35% (Lichtenstein et al., 2000), yet only up to 5% is associated with well‐characterized hereditary syndromes (Jasperson, Tuohy, Neklason, & Burt, 2010), which are caused by germline mutations in known high‐penetrance CRC genes (Al‐Tassan et al., 2002; Kinzler et al., 1991; Lynch & de la Chapelle, 2003; Palles et al., 2013; Weren et al., 2015). A substantial proportion of CRC syndromes have been associated with malfunctioning DNA repair pathways (Al‐Tassan et al., 2002; Hendriks et al., 2006; Nicolaides et al., 1994; Peltomaki, 2001). Recently, an autosomal dominant CRC syndrome, caused by monoallelic germline mutations in the exonuclease domains of *POLE* (OMIM #174762) and *POLD1 *(OMIM #174761)*,* was identified. Palles et al. reported heterozygous germline variants in the exonuclease domains of the DNA polymerases *POLE* and *POLD1. *These variants predispose individuals, with a high penetrance, to multiple colorectal adenomas and early‐onset CRC. These mutations were found by whole‐genome sequencing and linkage analysis in three large families that each possesses a dominant pattern of CRC and multiple adenomas. Subsequent screening of 3,805 CRC patients revealed that these variants are relatively rare in patients with a positive family history of adenomas or CRC: *POLE* p.(Leu424Val) was found 12 times and *POLD1* p.(Ser478Asn) only once. The tumors from the carriers were microsatellite stable and showed a hypermutated phenotype (Palles et al., 2013). Further studies have shown that *POLE* and *POLD1* mutations predispose individuals to multiple adenomas and early‐onset CRC (Bellido et al., 2016; Chubb et al., 2015; Elsayed et al., 2015; Esteban‐Jurado et al., 2017; Rohlin et al., 2016; Spier et al., 2015; Valle et al., 2014). The germline *POLE* p.(Leu424Val) mutation was demonstrated to also be associated with a Lynch syndrome‐like phenotype with microsatellite instable (MSI) tumors and somatic MSH6 or MSH2/MSH6 protein loss in the tumors (Elsayed et al., 2015). Additionally, *POLE and POLD1* somatic mutations can give rise to a Lynch syndrome‐like phenotype and microsatellite instable colorectal cancer (Jansen et al., 2016). To discover the underlying genetic causes of multiple colorectal polyps and CRC in genetically unexplained cases, we aimed to screen the exonuclease domains of *POLE* and *POLD1* in this group of patients. Generally, identification of new heritable risk factors may play a role in increasing the understanding of mechanisms underlying multiple polyp initiation and assist in the implementation of preventive strategies.

## 2 MATERIAL AND METHODS

### 2.1 Samples

Ethical compliance; the study was approved by the local medical ethics committee of the Leiden University Medical Center (P01–019). A total of 332 index patients with multiple colorectal polyps were included in this study. All patients were originally referred to the Laboratory for Diagnostic Genome Analysis in Leiden for possible FAP or MAP syndrome; no potentially pathogenic germline variants were found in the entire genes *APC* (OMIM #611731) and *MUTYH (*OMIM #604933). More recently, the patients had tested negative for *POLE* NM_006231.2:c.1270C>G, p.(Leu424Val) and *POLD1* NM_002691.3:c*.*1433G>A, p.(Ser478Asn) variants (Elsayed et al., 2015) and for *NTHL1* (OMIM #602656) NM_002528.6:c.268C>T, p.(Gln90*). Clinical data were collected from the Netherlands Foundation for the Detection of Hereditary Tumors (NFDHT) and from clinical genetics departments in the Netherlands; collected data included date of birth, gender, date of diagnosis with polyps, cumulative number of polyps counted at colonoscopy or in excised bowel, location and histology of polyps, the presence of duodenal polyps, information on CRC, presence of polyps/CRC in first degree family members, date of last contact and status at last contact.

### 2.2 Targeted next‐generation sequencing

Targeted next‐generation sequencing of leukocyte DNA was performed using a custom M13‐tailed sequencing panel on the Ion Torrent platform (Thermo Fisher, Waltham, MA). The exonuclease domains of *POLE* exons 9–14 and *POLD1* exons 8–12 were screened. Primers for overlapping amplicons were designed using Primer3 (http://primer3.ut.ee/) and ordered from Integrated DNA Technologies (IDT Leuven, Belgium). Primer sequences are available upon request. Following the manufacturer's protocol ‐ briefly, PCR amplicons were generated from 10 ng of leukocyte DNA using two primer pools. The PCR pools were subsequently combined and purified using AMPureXP beads. To add sample barcodes and Ion Torrent adapters, a second round of PCR was performed using M13 primers with A and P1 tails and sample barcodes. The PCR products were pooled, purified using AMPureXP beads and quantified using the Bioanalyzer High Sensitivity DNA kit (Agilent Technologies, Santa Clara, California). Size selection was performed, and the final concentration of the library was measured with a Bioanalyzer High Sensitivity DNA kit. Emulsion PCR was performed on an Ion One Touch 2 System (Thermo Fisher). The quality of the emulsion PCR was measured using the Qubit IonSphere Quality Control Kit, and libraries were sequenced using the Ion Personal Genome Machine (PGM).

### 2.3 Data analysis

The sequence data were checked for quality using the quality control tool for high‐throughput sequence data, FastQC (http://www.bioinformatics.babraham.ac.uk/projects/fastqc/). Subsequently, data were aligned to the human genome 19 (hg19, Genome Reference Consortium GRCh37) as a reference using the Burrows‐Wheeler Aligner (BWA, http://bio-bwa.sourceforge.net). Variant calling was performed using VarScan software (http://varscan.sourceforge.net/). Subsequently, variant annotation was performed with Annovar software (http://annovar.openbioinformatics.org). Variants with a minor allele frequency (MAF) >1%, as reported in dbSNP, ExAc or Go‐ESP, were also excluded. The Integrative Genomics Viewer (IGV, http://software.broadinstitute.org/software/igv/) was used to visualize the read alignment and the presence of variants against the reference genome. Alamut software (Interactive Biosoft‐ware, Rouen, France), Align GVGD (http://agvgd.hci.utah.edu/agvgd_input.php), PolyPhen‐2 (http://genetics.bwh.harvard.edu/pph2/) and Combined Annotation Dependent Depletion (CADD, http://cadd.gs.washington.edu/snv) were used for variant interpretation.

### 2.4 Validation and segregation analysis by Sanger sequencing

Sanger sequencing was performed to validate the *POLD1 *NM_002691.3:c.961G>A, p.(Gly321Ser) and *POLD1* NM_002691.3:c.955 T>G, p.(Cys319Gly) variants detected by the next‐generation sequencing panel, followed by co‐segregation analysis for available material from family members. Leukocyte DNA, in addition to both normal and tumor DNA, was used when available. Sanger sequencing was performed by Macrogen (Amsterdam, the Netherlands). Sequencing results were analyzed using Mutation Surveyor software (Sofgenetics, State College, PA).

## 3 RESULTS

A cohort of 332 Dutch patients with multiple colorectal polyps, without known pathogenic germline mutations, were screened to identify mutations in the exonuclease domain of *POLE *and *POLD1*. The mean age at diagnosis of colorectal polyps was 55.48 years (range 13–82). Approximately 44.9% of the patients have adenomatous polyps, while 43.3% of the patients displayed a mixed phenotype, predominantly adenomas with hyperplastic or serrated type. The majority of cases (56.6%) had a cumulative polyp count of 10 to 50. CRC was found in 126 patients (38%) at a mean age of diagnosis of 53 years (range 21–80). Clinical characteristics of the index patients are summarized in Table 1. Using targeted next‐generation sequencing, we screened the exonuclease domain of *POLE* and *POLD1*. Two *POLE* NM_006231.2:c.1270C>G, p.(Leu424Val) mutation carriers that we previously reported (Elsayed et al., 2015) were included as controls in this study. We detected the *POLE* c.1270C>G variant in the controls, but no additional *POLE* mutations were found. For *POLD1*, we identified two variants. A heterozygous germline *POLD1* NM_002691.3:c.961G>A, p.(Gly321Ser) variant located in the exonuclease domain (EDM) was identified in patient P1. In silico analysis predicted that this variant is likely to affect the function of the protein. The amino acid is highly conserved across species, up to Baker's yeast, and highly conserved at the nucleotide level (PhyloP: 5.53). There are small physicochemical differences between glycine and serine (Grantham distance: 56 [0–215]). Although the glycine and serine differ in polarity, charge and size, this change is considered a conservative amino acid substitution. This variant is predicted to be deleterious (SIFT score: 0.0), disease‐causing by Mutation Taster (*p*‐value: 1), possibly damaging by PolyPhen‐2 v2.2.2r398 (score of 0.88 [sensitivity: 0.82; specificity: 0.94]), and likely to interfere with function by Align GVGD (class C55 [GV: 0.00 ‐ GD: 55.27]). Furthermore, the Combined Annotation Dependent Depletion (CADD Phred, v1.3) is 29.7, predicting that this is may be pathogenic variant (Table 2). Patient P1 was diagnosed with multiple colorectal polyps at age 35 (>100 polyps, mostly hyperplastic and some adenomas) and a microsatellite stable (MSS) CRC at age 37. In addition to the *POLD1* variant, the patient is heterozygous for the pathogenic NM_001128425.1:c.536A>G, p.(Tyr179Cys) variant in *MUYTH. *The patient has no known family history of CRC; furthermore, no analyzable tumor tissue is available for further study.

**Table 1:** Clinical characteristics of the index patients included in this study (*n* = 332)

Clinical characterization	Individuals %	
Number of polyps		
<10	53 (16.0%)	
10–50	188 (56.6%)	
50–100	49 (14.8%)	
>100	29 (8.7%)	
Unknown	13 (3.9%)	
Type of polyps		
Adenomas	149 (44.9%)	
Adenoma + hyperplastic	103 (31.0%)	
Adenomas + hyperplastic + serrated	32 (9.6%)	
Adenoma + serrated	7 (2.1%)	
Hyperplastic + serrated	2 (0.6%)	
Hyperplastic	5 (1.5%)	
Serrated	1 (0.3%)	
Unknown	33 (9.9%)	
Age at diagnosis with polyposis		
>50 y	186 (56.0%)	
≤50 y	146 (44.0%)	
Diagnosed with CRC		
Yes	126 (38.0%)	
No	206 (62.0%)	
Sex		
Male	191 (57.5%)	
Female	141 (42.5%)	

**Table 2:** *POLD1* germline variants in the exonuclease domain identified by next‐generation sequencing

Patient	Alteration in genomic DNA	Protein alteration	MAF	rsID	Mutation taster	SIFT	PolyPhen−2	Grantham distance	Align GVGD	Segregation	CADD	Variant classification
P1, P2	c.961G>A	p.Gly321Ser	ExAC=0.0005 Go‐ESP=0.0002 TOPMED=0.0003	Rs41554817	Disease causing	Deleterious	Possibly damaging	Predicted not to be deleterious	Likely to interfere with function	P1: Segregation not performed, unclear family history For P2: not segregate in tested family members	Predicted to be pathogenic	VUS
P3	c.955 T>G	p.Cys319Gly	N.A	N.A	Disease causing	Deleterious	Probably damaging	Predicted to be deleterious	Highly likely to interfere with function	Not segregate in tested family members	Predicted to be pathogenic	VUS

Note

MAF: minor allele frequency; rsID: variant identifier in dbSNP, ExAc: exome aggregation consortium; Go‐ESP: exome sequencing project; TOPMED: trans‐omics in precision medicine; N3009.A: not available; CADD: Combined Annotation Dependent; VUS: variant of uncertain significance.

GenBank reference sequence: *POLD1; *NM_002691.3

**Figure 1:**
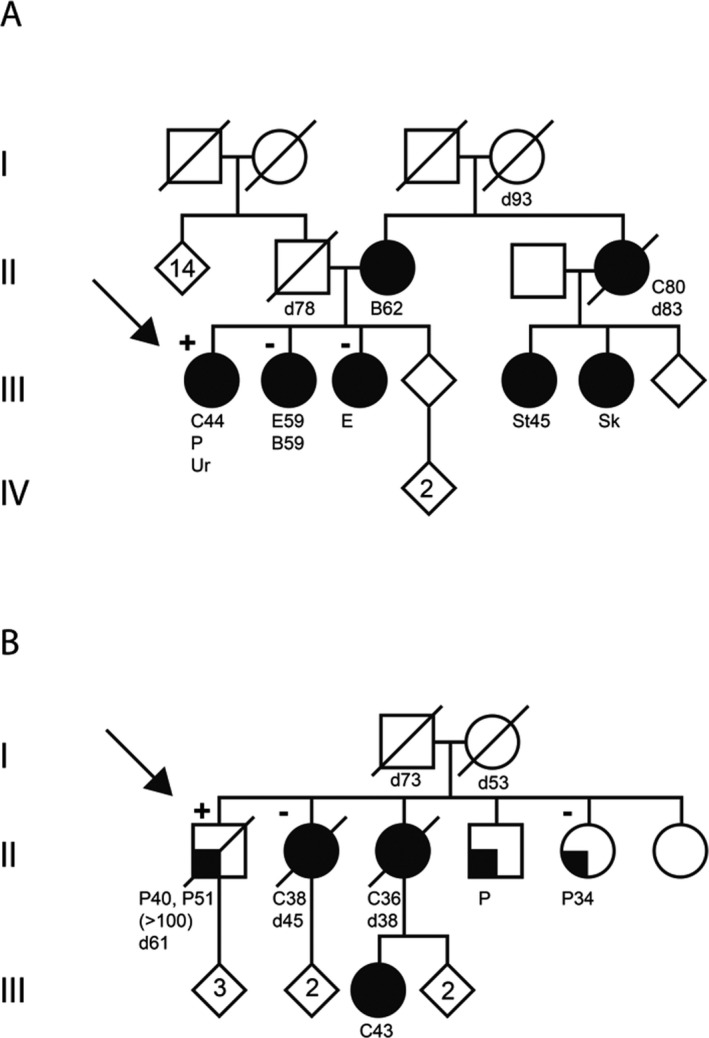
Pedigrees of the families with germline *POLD1* variants. a, represents the family pedigree of the index patient P2 with *POLD1* c.961G>A, p.(Gly321Ser). b, represents the family pedigree of index patient P3 with *POLD1* c.955 T>G, p.(Cys319Gly). Filled symbol, cancer; symbol filled quarter, individual with colorectal polyps. [+], *POLD1* variant carrier; [‐] noncarriers. The probands are indicated by an arrow. C, colorectal cancer; P, colorectal polyps; Ur, urothelial cell cancer; E, endometrial cancer; B, breast cancer; St, stomach cancer; Sk; skin cancer; d, deceased; number next to letter, ages at diagnosis or at death

Another patient P2 was identified with the *POLD1* c.961G>A, p.(Gly321Ser) variant. The patient was diagnosed with CRC at age 44 and one adenomatous polyp with low‐grade dysplasia at age 47 and two serrated adenomas at age 54. She had two sisters with endometrial carcinoma (EC). Both sisters did not carry the variant (Figure 1a). The tumor from this patient is mismatch repair deficient (MMRD), with microsatellite instability (MSI‐H), negative MLH1/PMS2 immunohistochemistry and with *MLH1* promoter hypermethylation (OMIM #120436). No somatic mutations in *KRAS *(OMIM #190070) exon 2, codons 12/13 and *BRAF* exon 15 (OMIM #164757) were found. Due to the highly degraded nature of the formalin‐fixed paraffin‐embedded (FFPE) derived DNA, we were unable to determine the tumor mutation burden.

The second *POLD1* NM_002691.3:c.955 T>G, p.(Cys319Gly) exonuclease domain variant was identified in a patient (P3) diagnosed with multiple colorectal polyps at ages 40 and 51 (>100 adenomas). Co‐segregation was performed using available DNA from affected family members. One sister cumulatively developed multiple polyps from age 34; however, leukocyte DNA tested negative for the variant. Another sister, diagnosed with CRC at age 38, had both normal and tumor DNA available for further analysis. Using both DNA samples, this case was shown to be a noncarrier. No further DNA was available for two other affected family members (Figure 1b). This variant could affect the function of the protein, the affected amino acid is highly conserved and the affected nucleotide is also moderately conserved (phylop: 2.87). With a large physicochemical difference between cysteine and glycine (Grantham distance: 159 [0–215]), the variant is predicted to most likely interfere with function of the protein by Align GVGD (class C65 [GV: 0.00–GD: 158.23]) and probably damaging by PolyPhen‐2 v2.2.2r398 (score of 1.00 [sensitivity: 0.00; specificity: 1.00]). The CADD Phred v1.3 score of 24.4, predicting that this is a pathogenic variant that is furthermore supported by SIFT (score: 0) and Mutation Taster (*p*‐value: 1) (Table 2). In summary, this variant is a novel missense change that might be damaging to protein structure and function but did not show convincing co‐segregation. No tumor material from the patient was available for further studies.

## 4 DISCUSSION

Pathogenic variants affecting the exonuclease domains of *POLE* and *POLD1* are associated with polyposis and colorectal cancer. Here, we screened the exonuclease domain of *POLE* and *POLD1* to detect causative variants in 332 index patients with multiple colorectal polyps. We detected two missense heterozygous variants in *POLD1*. The *POLD1* c.961G>A, p.(Gly321Ser) variant was identified in two patients with multiple colorectal polyps and CRC. Gly321Ser is highly conserved and predicted to be damaging by in silico analysis tools. However, the available evidence is currently insufficient to evaluate the effect of this variant on the function of the protein; therefore, the variant is classified as a Variant of Unknown Significance (VUS). Co‐segregation analysis was not feasible in one of the families and not supportive in the other family. In addition, the absence of available tumor tissue for functional analysis hampered further characterization of this VUS in P1. The tumor from patient P2 is MSI‐H with *MLH1* promoter hypermethylation, therefore the mismatch repair deficiency phenotype is caused by somatic *MLH1* promoter hypermethylation and not due to germline defects. While tumors from *POLE* and *POLD1* pathogenic variants carriers showed hypermutated phenotype (Elsayed et al., 2015; Palles et al., 2013), *POLD1* tumors with exonuclease domain mutations at highly conserved motifs (Exo1, 11, 111) were not consistently hypermutant (Campbell et al., 2017). It has been shown that mutations in *POLE* and *POLD1* do not always show a functional impact; therefore, determining the pathogenicity of mutations in these genes can be challenging (Campbell et al., 2017). The Gly321Ser variant is found in databases at a very low frequency (rs41554817, ExAc = 0.0005, GO‐ESP = 0.0002 and TOPMED = 0.0003). Interestingly, patient P1 also carries a heterozygous *MUTYH* p.(Tyr179Cys) variant in addition to *POLD1* c.961G>A, p.(Gly321Ser), possibly suggesting that both genes could act cooperatively and together confer an increased CRC risk. The co‐occurrence of the *MUTYH* pathogenic mutation with another mutation in *MSH2* or *MSH6* has been reported (Cohen, Tan, & Bisson, 2016; van Puijenbroek et al., 2007). Recently, a patient with the *POLD1* c.961G>A, p.(Gly321Ser) variant was reported; this patient developed CRC at age 41. No segregation analysis could be performed for the family as no DNA was available (Jansen et al., 2016).

The exonuclease domain *POLD1* c.955 T>G, p.(Cys319Gly) variant was identified in a patient who developed multiple colorectal polyps, with a family history of CRC and multiple polyps. The variant was only present in the patient but not in two affected siblings with CRC or multiple polyps, suggesting a possible de novo *POLD1* variant in patient P3. De novo mutations within *POLE* have been previously identified in some studies (Elsayed et al., 2015; Valle et al., 2014). While the in silico evidence suggests a pathogenic variant, the lack of co‐segregation in the family is not supportive. No tumor material from this patient was available to analyze further. However, it is still unclear whether or not the variant impaired protein function. Functional assays are required for better evaluation of these variants. Notably, the *POLD1* c.955 T>G, p.(Cys319Gly) variant was not observed in population databases (ExAc, GO‐ESP and TOPMED) and has not been reported in association with *POLD1*‐related disease. In the present study, we did not find pathogenic variants in *POLE* and *POLD1*. These genes have a low frequency in the predisposition for multiple polyps. It is worth mentioning that we previously evaluated the prevalence of the recurrent mutations *POLE *c.1270C>G, p.(Leu424Val) and *POLD1 *c*.1433G>A*, p.(Ser478Asn) in a cohort of Dutch index patients with multiple polyps or familial CRC. Although we did not detect *POLD1* p.(Ser478Asn), three index patients with the *POLE* p.(Leu424Val) variant were detected (Elsayed et al., 2015). Despite an enrichment in our cohort for inherited CRC and polyposis, the frequency (0.25%) is also comparable to reported frequencies (Palles et al., 2013; Spier et al., 2015; Valle et al., 2014). These results confirm the low frequency of these genes as a rare cause of the disease.

Recently, both *POLE* and *POLD1* mutations were identified outside of the exonuclease domains (Campbell et al., 2017; Esteban‐Jurado et al., 2017; Spier et al., 2015), suggesting other domains may be responsible for proofreading and should also be screened.

In conclusion, we identified no convincing pathogenic variants in exonuclease domains of *POLE* and *POLD1* in the current study. We recommend that screenings of *POLE* and *POLD1* should still be considered, although pathogenic variants in *POLE* and *POLD1* probably occur at a low frequency in patients with multiple colorectal polyps. Multigene panels offer significant benefits over sequential single‐gene testing by reducing costs, time and increasing the sensitivity. Moreover, making feasible the analysis of multiple low‐frequency genes in the highly heterogenous syndromes. Indeed, including the two genes in multigene panels that are used to screen for pan‐cancer mutations will allow to identify these rare mutations.

## CONFLICT OF INTEREST

The authors declare no conflicts of interest.
